# The Role of Field Training in STEM Education: Theoretical and Practical Limitations of Scalability

**DOI:** 10.3390/ejihpe10010037

**Published:** 2020-03-03

**Authors:** Kseniia Nepeina, Natalia Istomina, Olga Bykova

**Affiliations:** 1 Research Station of the Russian Academy of Sciences in Bishkek (RS RAS), Bishkek-49, RS RAS, Bishkek 720049, Kyrgyzstan; 2 Moscow State University of Geodesy and Cartography (MIIGAiK), Gorokhovsky pereulok, 4, 105064 Moscow, Russia; natali.istomina@mail.ru (N.I.); ol.p.bykova@gmail.com (O.B.)

**Keywords:** new educational model, higher education, skills, qualification, STEM education, geopolygon, test site, field training, Earth sciences

## Abstract

In this article, we consider the features of the perception of student information in science, technology, engineering, and mathematics (STEM) education, in order to draw the attention of researchers to the topic of learning in practice through field training. The article shows the results of these studies in Russia and the Commonwealth of Independent States (CIS countries: Armenia, Azerbaijan, Belarus, Kazakhstan, Kyrgyzstan, Moldova, Russia, Tajikistan, and Uzbekistan, as an example) to reflect the global trends. For this purpose, we examined the expectations of students in Russia and the CIS countries from training related to lectures and field training. We created a questionnaire and distributed it in three Moscow-based universities (Moscow State University of Geodesy and Cartography—MIIGAiK, Moscow Aviation Institute—MAI, and Moscow City University—MCU). Our key assumption is that field practices in Russian universities are qualitatively different from the phenomenon described in European literature, where digital or remote field practices have already emerged. The results obtained through the survey show the tendency of students’ perceptions to fulfill practical duties (in a laboratory with instruments of field training) in STEM education.

## 1. Introduction

The history of human society is divided into the three areas of its functioning—state and legislative, economic life, and cultural environment—through the development of which we can observe human history. The education system, i.e., the transfer of culture from generation to generation, is an essential element in the development of human society [1]. Global key trends and obstacles in the progress of higher education are connected by two facts. First of all, the personality of a contemporary student has changed. A separate structure has replaced the transition from homeschooling (parental) education. Second, educational technologies have been altered too. Due to knowledge transfer, nowadays there is an increasing number of technologies in higher education, and teachers can be trained in these new presenting methods. The specifics of studying science, technology, engineering, and mathematics (STEM) require mandatory support from theoretical courses with unique practices at training polygons.

Another change in the field of education that can be discovered from observations of the history of the development of human society is the introduction of the state–legal side to the cultural side of society. Initially, education was transmitted through the family. Then, for example, in England, in the 18th and 19th centuries, schools were a matter of social initiative. Nowadays, the ideas of economic life penetrate the education system; education is transferred to the service sector, while the state and legal influence on it does not wane. This position leads to opposite points of view, listed in [2]. That is why, currently, a person is considered uneducated without a state-approved document, and in the context of globalization, this trend is becoming global [3]. Unification of programs is appearing, education standards are being revealed, and the degree of uniformity in the transfer of knowledge is growing. Thus, more formal specialized methods for knowledge transfer must be forced to change. This is linked with the solution of using psychological and pedagogical tasks, as well as utilizing soft skills (teamwork training, leadership development, etc.). 

Now, we are witnessing the separation of intellect from personality. Industry 4.0 proclaims automatization that exalts the organization’s intelligence in the decision-making system, while the managerial role of the person is excluded. Personal changes are manifested in modern users of education. They differ from their predecessors in pragmatism, a sense of independence, lack of concentration of attention and inability to listen, clip thinking, and the need for self-expression [4]. Also, one of the most required abilities is to do patient and accurate fulfillment of similar tasks, which vary by visualization type.

The personal contemporary portrait of individual students is changing, as evident in the separation of the human “me” from nature, the appearance of a landscape in painting, the birth of nation states, the appearance of abstract thought in mathematics, and formal laws. However, this is not a complete list of the phenomena that indicate a change of eras, or the onset of the era of the new time. Such external conditions correspond to their own set of teaching aids for knowledge transfer: textbooks, learning aurally, or knowledge transfer using language concepts when using lectures. Note that the digital model of education does not scale to the study of scientific subjects related to the study of the history, evolution, and structure of the Earth.

Changes in the environment, including those due to the globalization process, the use of new tools such as online courses, and most importantly, a shift in personality, require a change in learning tools and methods. For learning, you need to use a different set of teaching aids: clips, teaching through visual images, knowledge transfer using new language concepts, replacing lectures with forms of independent work, followed by giving the student the opportunity for public expression to gain approval from members of the social group, but not the teacher. At the same time, it is precisely at the student age that the possibilities of perception, attention, memory, and thinking reach their maximum [5]. 

The most significant changes are currently taking place in the Eurasian space since the process of changes takes place in the context of the formation of new states and is inextricably linked with the creation of national education systems with new values. To date, this is the Consortium, whose partners are 16 leading universities in Russia, Belarus, and Ukraine, as well as Kazakhstan, Kyrgyzstan, Tajikistan, Moldova, and Armenia. The digitalization of the Russian and The Commonwealth of Independent States (CIS: Armenia, Azerbaijan, Belarus, Kazakhstan, Kyrgyzstan, Moldova, Russia, Tajikistan, and Uzbekistan) economy poses more challenges and prospects for the implementation of new educational technologies. 

It turns out that many students cannot master a specialty by only using visual images. The specifics of the disciplines related to the study of the Earth’s structure presupposes the mandatory follow-up of theoretical courses by particular training at the test site, which helps to neutralize the negative factors of indoor schooling. Knowledge could be improved during training when students solve a specific task in the field. Furthermore, the interaction between the teacher and students at the test site helps to resolve those problems that may not be solvable in the classroom or with online training (the formation of practical work skills goes along with the solution of psychological and pedagogical issues, the study of teamwork, and the creation of leadership qualities). It is necessary to satisfy students’ expectations from training related to lectures and field training. Afterwards, we can compare them with real trends in the dynamics of changes in educational technologies. Since these trends are global, it is necessary to identify the differences that distinguish them in the study of STEM. The purpose of this article is to show the results of experimental studies in Russia and in CIS countries in order to reflect the global nature of trends and the scalability limitations of modern educational technologies on the disciplines of studying Earth sciences. The work consists of an experimental study of the representations of students of specialties related to Earth Sciences, practical skills in STEM education. However, we still mention, as highlights, the problem of real STEM education training in terms of improving the quality of public management in the digital model of education. This article highlights today’s point of view in the academic environment; significance is placed on the non-European pedagogical model, in which the novelty lies inside the real analysis of student requests, although some European countries (for example, Latvia (www.lu.lv/en); Germany, University of Applied Sciences of Neubrandenburg, Albania, Serbia) and Latin America (for example, Mexico) also fit well into this system. 

## 2. Methodology 

The general forms of knowledge transfer have not changed. On the one hand, more formal specialized methods for knowledge transfer should be forced to change. On the other hand, a change in the personality structure of a contemporary person also requires an alteration in the methods of knowledge transfer from a teacher to a student. Although, the considerable flow of information and the need for its processing complicates the process of growing up, and young people remain infantile for much longer. Therefore, it is difficult to talk about a deliberate choice of a profession or about an uncompromising attitude to study. Thus, we consider the educational environment in Russia and the CIS countries (Armenia, Azerbaijan, Belarus, Kazakhstan, Kyrgyzstan, Moldova, Russia, Tajikistan, and Uzbekistan) to highlight global trends regarding the features of the perception of student information in STEM education.

**Figure 1:**
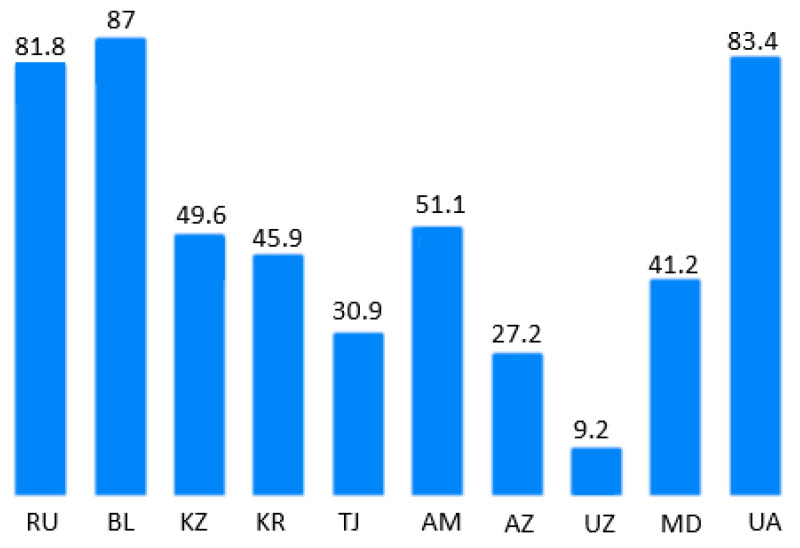
Percentage of the population getting the education in CIS countries (RU—Russian Federation, BL—Belarus; Republics: KZ—Kazakhstan, KR—Kyrgyzstan, TJ—Tajikistan, AM—Armenia, AZ—Azerbaijan, UZ—Uzbekistan, MD—Moldova, UA—Ukraine) in 2018 after [6].

First of all, we are interested in the proportion of the population getting an education in Russia and CIS countries. It is obvious (Figure 1) that three countries, in particular, have the most significant percentages of educative mass—Belarus, Ukraine, and Russia. 

**Figure 2:**
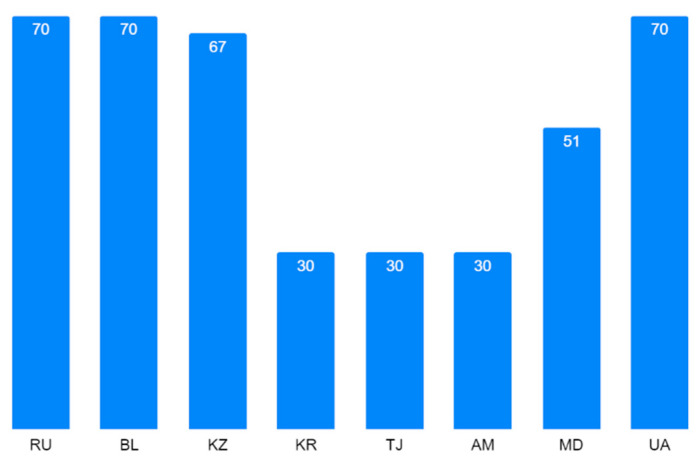
Percentage of the population speaking Russian (RU—Russian Federation, BL—Belarus; Republics: KZ—Kazakhstan, KR—Kyrgyzstan, TJ—Tajikistan, AM—Armenia, AZ—Azerbaijan, UZ—Uzbekistan, MD—Moldova, UA—Ukraine) in 2018 after [6].

**Figure 3:**
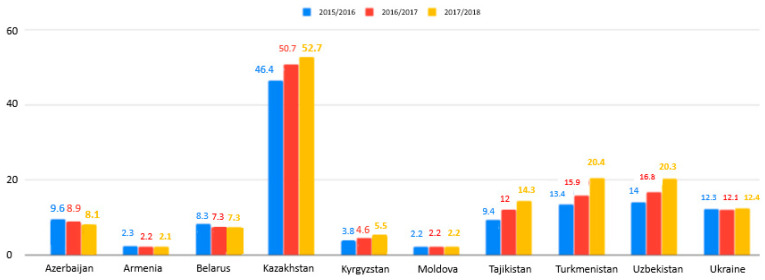
Students from the CIS countries studying in Russian state and municipal higher education institutions and scientific organizations with bachelor’s, specialist, and master’s programs in terms of standard admission at the beginning of the academic year (thousand persons) [6].

Learning in foreign languages opens up possibilities to study internationally. In [6], a list was given, showing the CIS ranging by English speaking skills between 80 different countries: Russia was in 38th place, Ukraine in 47th place, Azerbaijan in 64th place, and Kazakhstan in 67th place. Therefore, there is quite a low possibility to teach and to integrate into a globalization context. For Russia and CIS, Russian is the prime language (Figure 2). That is why the number of students from CIS countries studying in Russian state and municipal higher education institutions and scientific organizations with bachelor’s, specialist, and master’s programs in terms of standard admission has been increasing each academic year since 2015 (Figure 3): It was 124 K in 2015/2016, 132.7 K in 2016/2017, and 145.2 K in 2017/2018 in total.

Note that higher STEM education programs at Russian Universities tend to be taught in English. For example, St. Petersburg Mining University (https://spmi.ru) and Moscow Gubkin University (https://www.gubkin.ru) have been realizing such programs for a few years now. STEM education is in high demand in the mining and geophysics industry. However, it is not a popular program for the students, as the plentiful number of applied tasks frightens young and inexperienced students. In fact, in common with field training, the studying year passes with no summer holidays, and therefore, STEM education in Earth Sciences (which means subjects such as: geography, geodesy, geoecology, geotechnical engineering, geodesy, geomorphology, geology, and geophysics) is rather rare.

### 2.1. The Place of STEM Education in Russian Universities

According to the Russian Education portal, bachelor students of Geography study at 54 universities and master students study at 31 universities. In recent years, on a federal budgetary basis, about 1.4–1.8 K students were enrolled at the bachelor level in Geography, and 970–3400 students were enrolled at the master level (admission to the magistracy was significantly increased). Fourteen universities are currently preparing bachelor’s programs of hydrometeorology; master’s programs in this direction have been opened at 5 universities. General admission to the bachelor’s program includes more than 500 students, and to the master’s program, about 540 students. General admission to the bachelor’s degree in Applied Meteorology does not exceed 380 students, a set of undergraduates—about 400 students. Bachelor-cartographers study at 13 universities, and masters at 3 universities. The admission quota to the bachelor’s degree in Cartography and Geoinformatics is about 490 students. Herein, higher geographic and environmental education means the preparation of bachelors and masters in the fields of Geography, Hydrometeorology, Cartography and Geoinformatics, and Ecology and nature management. This direction is the only one in which the number of budget places for undergraduate studies has increased in recent years. In the course Ecology and Nature Management, bachelor students graduate in 168 and masters in 75 universities. Admission figures are compiled by the Ministry of Natural Resources and Ecology. In terms of quantitative training indicators for student geographers and ecologists, Russia lags behind many countries. For example, in the UK, geography is studied in more than 80 universities (the number of undergraduate geography students is 30 K); in Germany, in 60 universities; and in France, in 178 universities. In the USA, a bachelor’s degree in geography can be obtained at more than 200 Universities (annual graduation is more than 6 K), a master’s degree in geography in about 90 universities, and a doctor of geography (Ph.D.) in 60 universities. In India, an average of 12.5 K students study annually in undergraduate programs in geographical programs, 4–5 K students are enrolled in graduate and postgraduate studies annually, and these numbers are planned to increase significantly by 2020 [7].

The real state of education in STEM with Pedagogical Examples is given in [8]. Navigation skills and STEM learning, specifically related to the field of Geography and Geoscience (Earth Sciences), are essential as they help to pursue professional development. The ability to integrate and connect different routes to create a map of the environment, to relate them to each other, and to investigate them is a specialty of field training. From this point of view, research examining the effectiveness of geoinformation systems (GIS) training is under consideration [9]. “A geophysics class may also incorporate labor field-based activities using a particular instrument (e.g., radar, gravimeter) to measure variations in material properties, and then discuss potential limitations in the measurements that aid interpretations of the data. With both types of activities, developing that capacity to make logical conclusions that flow from the available data is a key focus of the learning experience” [10]. 

### 2.2. Field Training

The basic principle of practical training is a combination of practical exercises with simulation methods in the development of practical skills and fluency with new equipment. Field training is one of the main activities to ensure the development of hard skills. Field training in STEM education is much more effective because it connects both types of skills: soft and hard. The collective work using professional devices, for example, a surveying instrument with a theodolite (a rotating telescope for measuring horizontal and vertical angles), is usually made by at least two persons together. 

Moscow State University of Geodesy and Cartography (MIIGAiK) educates students in the scope of problems of geodesy, cartography, and cadastre, as well as such specific fields as precise instrument-making, geoinformatics, ecology, and remote sensing [11]. The glorious past of MIIGAiK, deep-rooted in pedagogical and scientific traditions accumulated throughout the 225 years of its development, the importance and vitality of geodetic science and practice for many branches of national economy, with a wide range of specialists being trained at the University—all of these factors assure the leading role of MIIGAiK as a specialized institution of higher education [12]. More than 2000 foreign students have graduated from the University. The University study provides theoretical and practical training, and STEM education disciplines (such are geographic information technologies (GIS)) are educated at the Department of Cartography of MIIGAiK [11,12,13].

The evidence that has emerged from the studies [14,15,16,17,18] has proved that the field-based approach is effective in helping students to attain the required knowledge for the understanding of the natural context, and to illustrate phenomena and confirm their skills. As well as soft skills, other competencies such as scientific reasoning and inquiry capacities are also developed. Training offers the opportunity to work as part of a team (as you know, this particular skill in recent years has been a weak spot among students of MIIGAiK, for example, as teachers who travel to practice with students have repeatedly stated). Students also learn safety rules, which are more crucial than in general conditions in classrooms. 

Another critical difference between domestic and European geographic education is that, in Russia, many more hours are devoted to training and production practices [19,20], particularly in undergraduate studies, with up to 36 h, which is 9%–15% of the total complexity. In Europe, the most widespread field practices are preserved at universities in the Netherlands (4 weeks), and in the UK, France, and Sweden, which do not exceed two weeks. During training, students travel not only to various regions of their country but also to non-CIS countries (for example, Africa, Central America, etc.). In most European universities, field practices are study tours lasting 1–2 days, the purpose of which is to teach students to work on a specific topic in small groups. Such trips are usually paid for by the students themselves; in an academic year, there are only 2–3 trips [7]. 

In Russia, in recent years, the problem of financing field training practices has come to the fore, especially in regional universities [7]. There is the threat of losing rich experience in conducting field training as the practice, which has always been not only a way to acquire and consolidate knowledge, but also a form of preparation for professional activity. In addition to funding problems [19], Russian universities are faced with organizational issues in conducting training and production practices, in their methodological and instrumental support, and in being equipped with modern laboratory equipment. 

Principally, training in STEM education has a number of disadvantages. “There may be gendered issues, cultural and language barriers, logistical issues, security issues and problem in creating accurate risk assessments without prior visits by staff. There are also issues around privileged, educated, and relatively affluent university students going to view and study underprivileged groups or locations in poorer societies, often without their consent” [20]. There is a potentially high risk for safety violations because of weather conditions. Virtual reality (VR) classes could be limited by technology and have struggled to expand due to a lack of computing power and memory [21].

Education is mostly impacted by new spatial visualization software and virtual reality (VR) in higher education, which is a big trend [21]. Also, in some places, geological virtual field experiences (VFEs) have been developed that consist of form high-resolution two-dimensional (2-D) photomosaics and three-dimensional (3-D) computer models [10]. VFEs can potentially eliminate some of the issues that physical field trips can create.

According to new educational technologies, some countries use Virtual reality techniques, such as in [8,10,15,16,17,18,21,22,23,24,25,26,27,28,29,30,31,32]. This is because the students need visualization in order to provide evidence of some geological processes, for example, plate tectonics. A comparison of the most popular models of head-mounted display systems for virtual reality (VR) used for educational purposes is given in [10]. However, we agree with [10] that sometimes, some test scenarios would be too difficult or dangerous to perform in real life.

Based on the analysis of some institutional changes in the higher education system, we aim to justify the conclusion that the problem of obtaining knowledge in real space in the digital age is growing. Comparing the collected data with descriptions of the problem in foreign literature, we show that the domestic higher education system is characterized by a particular type of student dropout, associated with physical limitations in the admission of students, which requires individual study and targeted measures of educational policy in connection with the characteristics of the profession. At the moment, even if there is no possibility of an internship within the university, students are forced to undergo field practices outside the university. That is why it is so essential to create and maintain the stability of field test training sites or geopolygons. The requirements for geopolygons are restricted. First of all, remoteness from the noise is needed. Second, pure nature is essential in order to conduct experiments (such as drone test). Field scientific and educational centers will provide cooperation of the educational, scientific, and production organizations in the sphere of Earth sciences. They will be based on the principle of unity of technologies of scientific expedition support [19]. As such, the Research Station of the Russian Academy of Sciences at the Bishkek Geodynamic polygon is suitable for these conditions [33,34,35]. The network of field test sites will include regional field scientific and educational centers. At MIIGAiK University, there is a Site called Chekhov Geopolygon [36]. Moscow State University established a geophysical training site 200 km from Moscow on the Russian platform in 1992 [37,38,39]. The development of field test sites and saving the key long-term field reference areas are both possible as part of a national network. The unified standard infrastructure of field test sites will allow the efficient scaling and distribution of modern methods of researches in expeditions. The optimization of a network of field test sites is possible on the basis of their inventory, determination of their uniqueness, status, and perspectives of development [19].

### 2.3. STEM Online Education at the National Universities

Massive open online courses (MOOC) is a new tool in education [40,41]. However, serious pedagogical concerns associated with the inability of an instructor to provide individualized instruction to thousands of students have emerged [42,43]. Also, according to [42], the analysis of the completion of the course from the “Satellite Technologies in Geography” section concludes that: “It is not true that a MOOC must necessarily be absent of any faculty presence, just as it is also not true that a successful MOOC must only feature lecture videos or be taught by a faculty member who is famous”. 

It should be noted that the grant support is aimed today at the digitalization of education “Modern Digital Educational Environment in the Russian Federation” [44]. The results are visible via digital platforms [45,46].

The online resource aggregator http://neorusedu.ru/ [45] provides access to courses from Russian Universities, expanding exclusively for Russian citizens the opportunity to study throughout their life at any convenient time, wherever they are at that moment—the main requirement for these types of courses is that an Internet connection is available. Additionally, this resource pays excellent attention to attracting the cooperation of employers. If the electronic certificate received after the final exam serves as a confirmation that students completed the test (more than a hundred universities are connected to the “one window” resource, whose students master some of the disciplines online), then for the rest of the students, it is a certificate of new knowledge and competencies. One of the educational models provides for the complete replacement of the discipline in the curriculum with online courses. The second educational model is that of blended learning, where online courses replace only the theoretical part of the subject. The practical part—laboratory, workshops, seminars—is accompanied by a university teacher. Today, educational organizations are more likely to lean toward the second model.

Another national platform of open education, “Open Education” https://openedu.ru/ [46], is a modern educational platform that offers online courses in basic disciplines studied at Russian universities. The Association “National Platform created the platform for Open Education” was established by the leading universities Moscow State University M.V. Lomonosov, St. Petersburg Polytechnic University, St. Petersburg State University, National University of Economics “MISiS”, Higher School of Economics “HSE, MIPT”, Ural Federal University “UrFU”, and Saint Petersburg National Research University of Information Technologies, Mechanics and Optics “ITMO”. All courses posted on the Platform are available free of charge and without formal requirements of a basic level of education. Compared to courses of other online learning platforms, courses of the national platform have certain features:

- all courses are developed following the requirements of federal state educational standards;
- all courses meet the requirements for the results of training of educational programs implemented in universities;
- special attention is paid to the effectiveness and quality of online courses, as well as to the procedures for evaluating learning outcomes.

The international Coursera portal (https://www.coursera.org/) contains programs from prestigious universities and employers from around the world, from Russian Moscow State University and St. Petersburg State University to Stanford University and Google [47]. More than 50 courses are now offered in Russian or with Russian subtitles. However, on international platforms, all courses are covered in English, while on national platforms, they are in their native language. However, we are faced with a problem: most online courses are aimed at first-year or second-year students.

As a procedure for understanding trends in STEM education in Russia and CIS countries, we examined the students’ expectations. As an instrument, we have used the online survey (see Table 1), which we have created in later 2019. The participants present three Moscow-based universities (Moscow State University of Geodesy and Cartography—MIIGAiK, Moscow Aviation Institute—MAI, and Moscow City University—MCU) and some academic staff. In the results section we provide data analysis.

## 3. Results 

The educational environment in Russia and CIS countries reflects the global world trends in learning processes. With the introduction of digital learning technologies, knowledge transfer has changed. Massive open online courses and open learning platforms are designed to show that distance learning can be scaled to all educational specialties. However, teaching technologies in the form of passive contemplation by students of the visual range and solutions of test problems cannot be scalable to study the disciplines necessary to obtain professional competencies and knowledge in STEM education (Earth sciences). STEM education specifically requires the mandatory support of theoretical courses with individual practices at the test sites. Inversely, we observe a decrease in the educational time units designed for training practices in STEM and Earth sciences (geography, geodesy, geoecology, geotechnical engineering, geodesy, geomorphology, geology, and geophysics) —there is a tendency to replace off-site activities with online and VR technologies.

In order to show the limited scalability of modern educational technologies to disciplines related to the study of Earth sciences, we give arguments confirming the importance of the formation of practical work skills, which goes hand in hand with the solution of psychological and pedagogical tasks, teamwork training, leadership formation, and patient and thorough implementation of the same tasks. Based on the results of an empirical study conducted in three Moscow universities, and including a survey of some students and some respondents who recently graduated from the university, we consider the peculiarities of students’ perceptions of information in STEM (Earth sciences) and their expectations from the learning process. We proceed from the fact that field practices in Russian universities are qualitatively different from the phenomenon described in the European literature, where digital or distance field practices for people with disabilities are already appearing. Thus, we developed a special online survey, with the idea to highlight the point of view of a modern student on educational technology.

The level of preparedness of students to work with electronic resources is very heterogeneous. Thus, the development of resources and Internet technologies comes to the fore. At the same time, there has been a rapid growth in information and communication technologies (video conferencing, webinars, groups on social networks, and many others), which makes it possible to organize not only the passive perception of online courses, but also the active remote interaction of students, a collective discussion of the material, and joint execution of tasks, all while at significant physical distances from each other. Therefore, the university teacher is now faced with the need to possess not only subject knowledge and modern technical means, but also management techniques of computer-mediated educational communication. Mastering the educational form of information education is an urgent task of our time.

The students’ lack of understanding of the concepts of a map section, a cross-section of a complex technical object (spatial ability into constituent spatial skills), and aberrations of an optical image of a star in a telescope observed by students moves us toward a change in the methods and technologies of teaching technical disciplines for students of geodesic, cartographic, and geophysical specialties from visual static illustrations to dynamic pictures as of films. To handle the material, it is necessary that when reading a lecture, an interactive way of conducting classes is used [6]. Such tools can provide access to the Internet and gadgets. American physicist Michio Kaku believes that the basis of future education is Google Glass, and those who study through lectures are losing out [20].

However, if in the case of training, the student is gaining courses following the curriculum, then such an instrument does not exist for the employee of the company (teacher, researcher, engineering, and technical personnel). In this case, the employee sometimes must forcibly acquire knowledge at any other possible sites.

**Figure 4:**
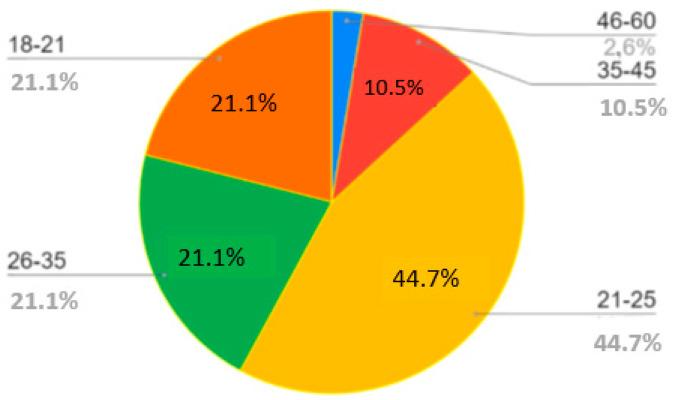
Survey results. Question 1. Age.

**Figure 5:**
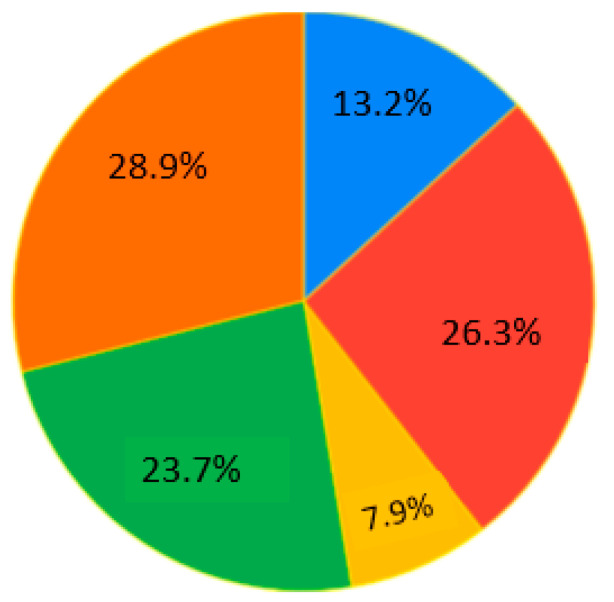
Survey results. Question 2. Occupation. Sectors: blue—employee, red—researcher, yellow—unemployed, green—master’s student, orange—bachelor’s student.

**Figure 6:**
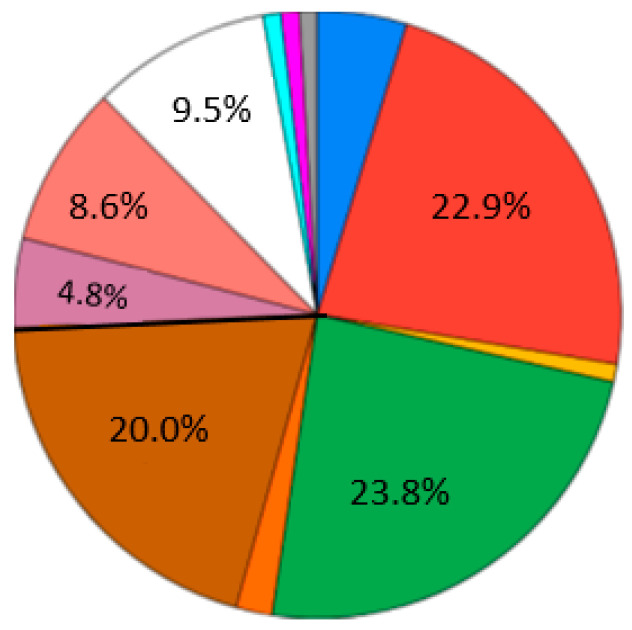
Survey results. Question 3. Study objectives. Sectors: blue—there are so many changes in the world that my skills quickly become obsolete (4.8%); red—obtaining skills for mastering a specialty; yellow—none, don’t know (1%); green—obtaining knowledge; orange—while there is no universal and suitable program for me, I have to study in different places; brown—I want to learn everything new; purple—I want to gain an additional specialty; pink—Social status and prestige; white—have a good time within the student auditorium; other (~3%), please specify: light blue—learning ability; magenta—opportunity to “find” interesting topics for professional development; grey—I need education. Development is interesting, I hope to get into an adequate structure, where you really learn and develop, and do not waste time talking and talking for the sake of reporting.

**Figure 7:**
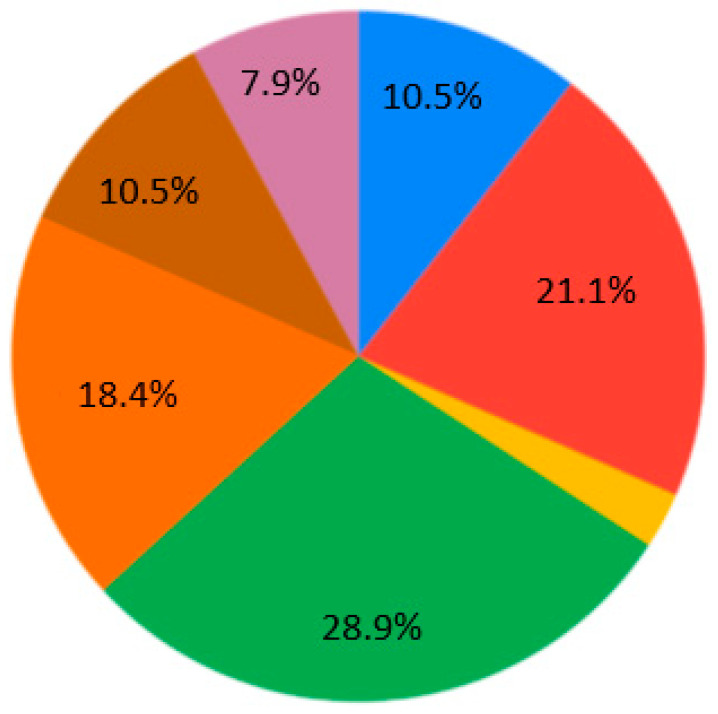
Survey results. Question 4. Lectures perception. Sectors: blue—independently (in the library, in the book); red—as presentations, communication with a lively person; yellow—I do not like to learn new material; green—solving case studies (a method of specific situations) or a game; orange—tête-à-tête with teacher/lecturer/mentor; brown—as a joint discussion as a type of crowdsourcing; purple—as lectures, the audience is communicating with a lively person. Nobody selected the answer “As lectures, communication via Skype, but with one person”.

**Figure 8:**
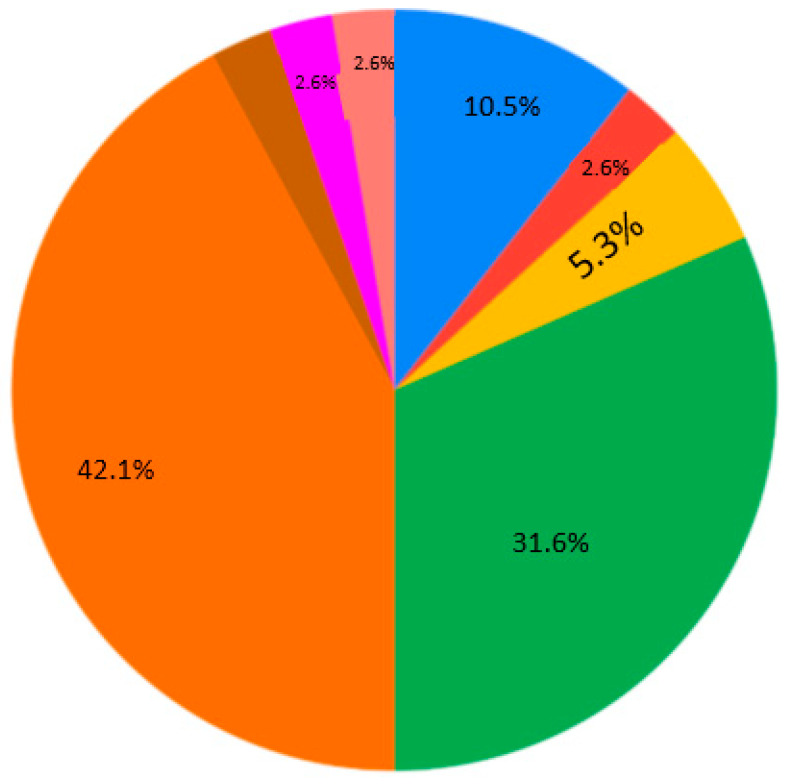
Survey results. Question 5. Lectures repetition. Sectors: blue—need to take and learn new topics and not to repeat; red—needs to be repeated if necessary; yellow—does not need to be repeated; green—needs to be repeated 1–2 times in the school year; orange—needs to be repeated 1–2 times per semester; answers added by the students (other): brown—regular and straightforward control of knowledge and study of the material helps a lot; purple—the most effective way to consolidate knowledge is to drive through various mechanisms of perception and reproduction by a person: listen, write something, try to convey it to someone; pink—needs to be repeated 1–2 times per semester through case studies, seminars, and lab classwork.

**Figure 9:**
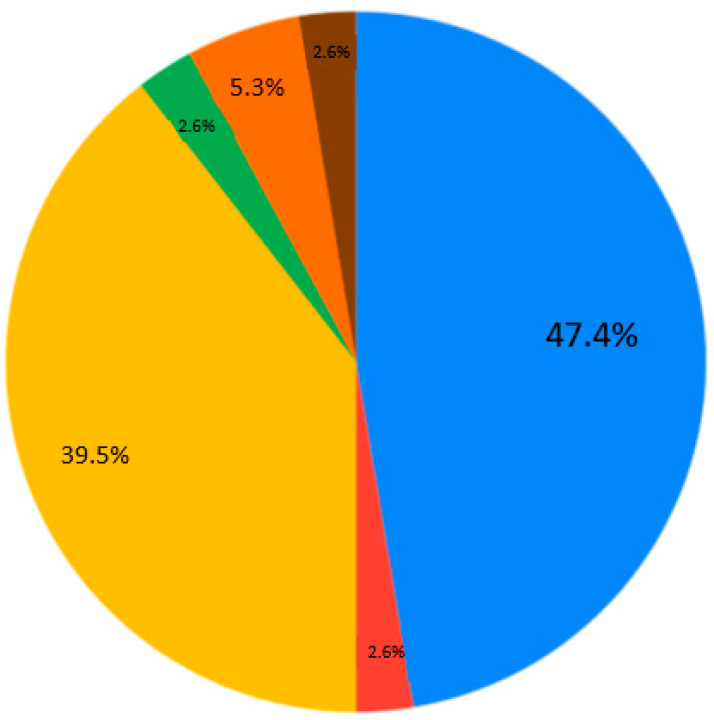
Survey results. Question 6. Proving skills. Sectors: blue—as practical work with geodevices; red—I do not need practical exercises to consolidate skills, as I remember everything; yellow—to create a specific project; green—cogitation tasks that can then be discussed with the teacher and other students, and not just tests; orange—as competition among students, during which my strengths and weaknesses are determined; brown—answers to tests via a computer, where my answers are checked by a robot.

**Figure 10:**
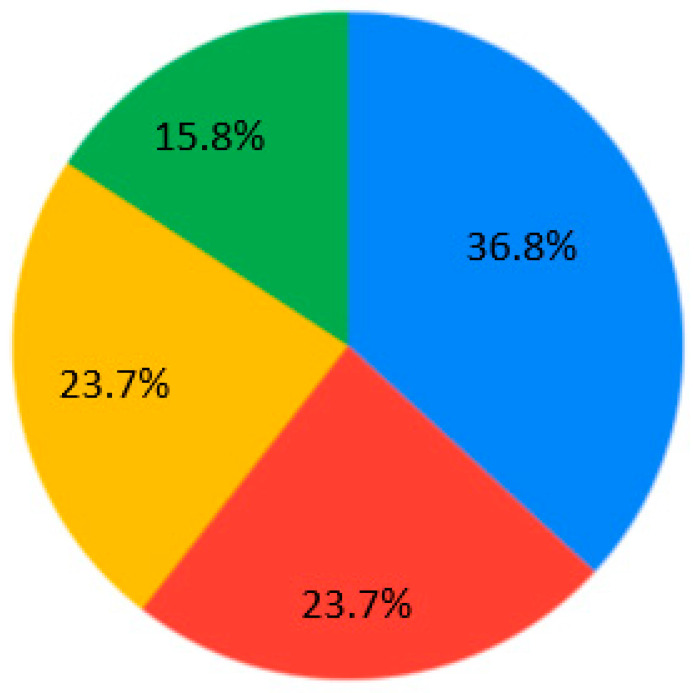
Survey results. Question 7. Visual displays. Sectors: blue—paper versions; red—computer screen; yellow—Virtual Reality headset; green—Movie Screen. The answer “I don’t need to watch. I remember aurally” was not chosen.

**Figure 11:**
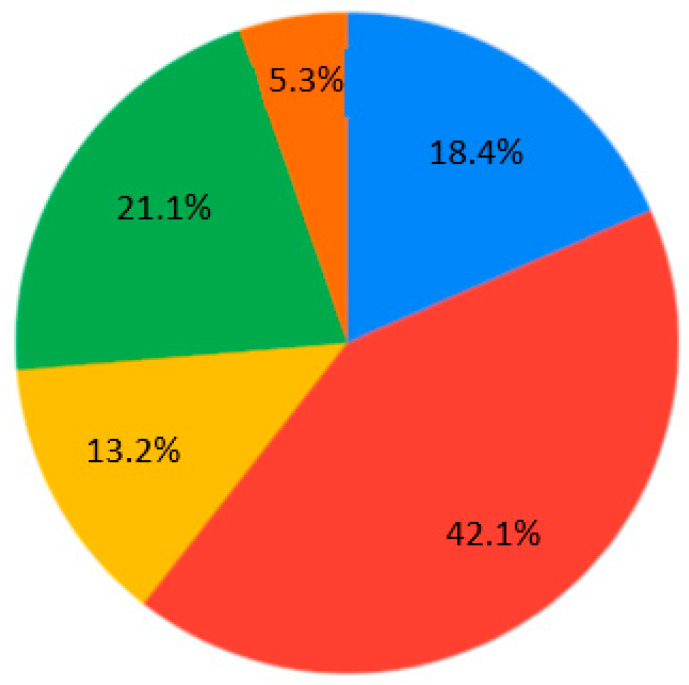
Survey results. Question 8. Lecture time. Sectors: blue—I can withstand more than 2 h if I am allowed to move, rather than just sitting in one place; red—45 min; yellow—lying down, I can listen as long as I like; green—1.5 h; orange—15–18 min.

**Figure 12:**
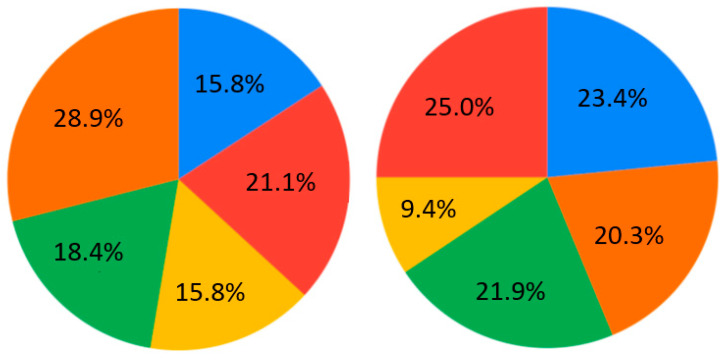
Survey results. Questions 9 and 10. Future work perspectives. Left—the first option, right—the second option. Sectors: blue—I like to travel and do field research; red—remotely as a freelancer; yellow—I do not want to work, I want to continue to study; green—in the office among other employees; orange—managing innovations.

In 2019, a voluntary Survey (details in the Table 1) about state-of-the-art education in the Geoscience Faculty of MIIGAiK, the faculty of Innovations and a pedagogical group of students answered questions. Short questionnaires were administered to 64 respondents, including students from the bachelor’s and master’s programs, with ages ranging from 18 to 25 (average age, 23 years). The observation reports were powered by the researchers (the authors). The advantage of resorting to diverse techniques and instruments to collect data was the possibility to triangulate quantitative and qualitative methods, thus enhancing the validity of the results. The validation of the short survey was carried out by the authors. The questionnaires that were administered to evaluate the preparatory and summary units had the following closed questions (Table 1), which had to be answered at least in 5 variants. Reports were written after the class to verify the relevant (positive and negative) aspects of the mediation process, the difficulties of the students’ engagement in the process, and an overall evaluation of the model that was implemented. Circle charts were used to group the items (see Figure 4, Figure 5, Figure 6, Figure 7, Figure 8, Figure 9, Figure 10, Figure 11 and Figure 12) and the description of results (below).

This is a description of the survey. It contains details and data supplemental to the main text. The main flow of questions is with answers (Table 1) using Google Forms online. We were oriented toward inclusive learning environments in undergraduate courses [48]. We are sure that such a kind of questionnaire provides new information on the current state of teaching practices in STEM education concerning inclusive practices. We expect to compare results in future projects. These results could lead to maintaining focused STEM education development. 

The self-reported use of practices across these four categories of ages (Figure 4) and five categories of occupation (Figure 5) are also highlighted. The most significant numbers of testimonials were provided by bachelor’s and master’s students aged 21–25. 

The largest proportion of respondents chose their study objectives as getting knowledge (23.8%), followed by obtaining skills for mastering a specialty (22.9%) (Figure 6). Note, that there is still a large number who want to study for learning everything new (20% in Figure 6). This means that education is still not only implied for getting a job. 

The contemporary generation of students is interested in case studies at the universities (28.9%) (Figure 7). Generally, nobody selected the answer “As lectures, communication via Skype, but with one person” for Q4 (Figure 7). Near equally, the presentation and real communication or tet-a-tet conversation with the teacher are preferable (Figure 7). Concerning material repetition, it is an individual aspect. Most of the students are ready to repeat some material 1–2 times per semester/school year (Figure 8). Regarding the survey results (Table 1), the students are encouraged to test their skills as practical work with geodevices (47.4%) or by creating a case study project (39.5%) (Figure 9). It is also important that, for Q7, the answer “I don’t need to watch. I remember aurally” was not chosen (Figure 10). Despite the implementation of digital technologies, the students gain new information mostly via paper versions (36.8%), and equally less on a computer screen or a virtual reality headset (Figure 10). Most of them are ready for 45-min lectures (42.1%) or 1.5-h lectures (21.1%) (Figure 11). The greater proportion of respondents in the future desire, firstly, to manage innovation (28.9%) or to work remotely as a freelancer (21.1%) (Figure 12). Therefore, it is necessary to think about why modern methods of education are aimed to educate specific skills to rear the ideal workers, while not all graduates are ready to work for specific enterprises. 

We obtained survey results that, accordingly, show students’ desires to fulfill practical activities (in a laboratory with field training tools) during STEM classes. We believe that these training practices will be taken into account in the future.

## 4. Discussion

This article notes that the education system is a part of the national culture that is formed under the influence of history, geography of the country, social and social conditions of life, and which depends on the national mentality and the psychological activity characteristics of both students and teachers alike. The authors conclude that there are some difficulties in the higher education conditions of the 21st century. The new digital era raises the problem of the discrepancy between the skills that a student receives online and the skills he must master during on-site field training.

We agree with Kuznetsov [41] that Russian students themselves are not ready to accept digital education as a fully-fledged learning method. “The correction of this situation requires the introduction of technological and organizational solutions in the field of education, aimed at adapting the educational system to the dynamically changing needs of the labor market, individualization of educational trajectories and increasing the involvement of students in the educational process”. There is a deficient percentage of students who complete online courses successfully. The teaching of speech disciplines in technical universities is unusual and does not involve everyday usage. Particular attention is paid to the interactive form of conducting classes.

It has been shown [41] that the education system, formed in the previous technological way, does not meet the needs of contemporary society. The main issue is that there still does not exist a system of recognition of the equality of online education in comparison to traditional forms of education. The fact that there is a continuing lack of implementation of online technologies in the educational process by educational organizations themselves impacts on the process speed. So, if today, in the enterprises and organizations of Russia, 50 personal computers are accounted for 100 employees, of which 33 are connected to the Internet, then in our universities, there are only 228 of these equipped workplaces. Against this fact, the general level of financing of educational organizations in Russia, which is 4.7% of the gross domestic product, seems insufficient. According to the accumulated statistics of the largest educational online platforms, the number of students who reach the end of the training is 5%–13% of the initial admission [41,49]. There are circumstances that highlight the lack of development of the digital infrastructure and the need to train teachers. STEM teachers should mix their identities as a teacher, learner, risk-taker, inquirer, collaborator, and inspector at the same time, especially during field training. Educational institutes need to take into account the difference in the pedagogical culture of international students and Russian teachers. Therefore, educational organizations should find more time and sponsor lecturer training too. 

Educational methodological approaches are directly related to existing technologies and the needs of students. However, on the other hand, they are under pressure from official directions of governmental control. Teachers always have to adapt to the available technology and the needs of the students in accordance with state strategy. In fact, we are talking about a change in the general model of higher education. Instead of a humanistic model, which interprets higher education as a human right to enrich oneself with the highest intellectual achievements of humankind and put them at the service of social progress, the economic model is legitimized, which treats higher education as a sphere of investment that brings additional income, financial dividends, and other bonuses—which is no longer consistent with the United Nations Declaration of Human Rights. Hence the direct path to the commercialization of relations between teachers and students, to the growth and the dominance of university management, to emasculating from university life everything that does not contribute to economic efficiency, i.e., profitability. The strategy of the digital economy in STEM education leads to a strong university bias toward scientific business services, the transfer of the center of gravity to paid custom research, which leads professors to devote most of their time to it because their status and well-being depend on their scientific activity. The situation emasculates from university life, everything that has little to do with applied tasks, especially with the preservation and development of social and art sciences.

## 5. Conclusions

The digital world is as essential and immersive as the real one. In a controlled environment, a pre-field trip can increase engagement in the topic studied. There are also benefits to the educator, such as reduced cost, more efficient students on fieldwork tasks, and the ability to tailor and update their field guides to suit their needs. However, there are drawbacks to the challenge of creation and their outcome as standalone educational tools. Online education will definitely develop, and at the same time, the skills of human communication will remain the most critical, which, as practice shows, is the most difficult to learn online. That is why we suggest that educational policymakers insights’ pay attention to and finance the outdoor learning environment as a potential obligatory activity for STEM education. A method of teaching using electronic learning management systems such as e-university and MOODLE can exist only as an addition to the usual forms of educational interaction with lectures and seminars. We agree with authors [20,21,41] that the tendency leading to a lack of teacher-student interaction through virtual reality technology is extremely scary. The potential balance between teaching hard and soft skills is a significant issue in the contemporary workplace. There must be an equilibrium between state-of-the-art solutions and human interaction, mentoring, and teacher–student relationships.

It is important to incorporate the outdoor learning environment as an integral part of the learning of STEM education. The best place to train geophysical or geonavigation competences is in well-prepared geopolygons with suitable infrastructure (such as internet access, remote low-noise area, well conditions, etc.). The teacher, having left the school department for the field training, is forced to change the official style of pedagogical communication into a familiar one, creating comfortable working conditions and increasing the effectiveness of learning outcomes.

Results section contains a description of the research methodology (survey, target group set, etc.). The study reveals that opportunities to be involved in practices are useful for students.

This article discussed the processes of global changes in STEM education of higher education, with flashbacks to Russia, linked to the acceptance of the Bologna declaration and digitalization, which is the outcome of global trends in higher education. This resulted, in particular, in the reduction of field training in STEM education. It also poses new requirements of lecturers in the system of higher education, and is involved in the process of new identity formation of being STEM teachers with the many roles and responsibilities associated with such an identity. Backlighted by active integration processes with international systems of education, it becomes evident that many Russian lecturers do not comply with the new requirements of the educational process: lack of or limited knowledge of foreign languages (English), lagging in “informatization” of education, mismatch in humanization and art science of the educational process. Thus, this article underlines the necessity for an anticipatory strategy in education, which is a crucial factor in the progressive development of the country.

## Figures and Tables

**Table 1:** Survey questions and answers (powered by Istomina N.L. and Nepeina K.S.).

No	Question	Answers
1.	Please indicate your age	● 18–21 years old ● 21–25 years old ● 26–35 years old ● 35–45 years old ● 46–60 years old
2.	Please indicate your occupation	● Bachelor student ● Master student ● Researcher ● Employee ● Unemployed
3.	Please indicate your study objectives	● Obtaining knowledge ● Obtaining skills for mastering a specialty ● There are so many changes in the world that my skills quickly become obsolete ● I want to gain an additional specialty ● Have a good time within the student auditorium ● I want to learn everything new ● While there is no universal and suitable program for me, I have to study in different places ● Social status and prestige ● other, please specify
4.	How is it convenient for you to perceive the new material?	● As lectures, where the audience is communicating with a lively person ● As lectures, with communication via Skype, but with one person ● As presentations, with a connection with a lively person ● As a joint discussion as a type of crowdsourcing ● I do not like to learn new material
5.	From your point of view, some materials/part of the lectures should be:	● need to be repeated 1–2 times per semester ● need to be repeated 1–2 times in the school year ● need to take and learn new topics and not to repeat ● Does not need to be repeated ● needs to be repeated if necessary ● other, please specify
6.	To prove your skills, you need:	● Answers to tests via a computer, where my answers are checked by a robot ● To create a specific project ● Competition among students, during which my strengths and weaknesses are determined ● Cogitation tasks that can then be discussed with the teacher and other students, and not just tests ● Practical work with geodevices ● I do not need practical exercises to consolidate skills, as I remember everything
7.	If you have the choice of training with visual equipment, then you would prefer:	● Computer screen ● Movie Screen ● Virtual reality headset ● Paper version ● I don’t need to watch, I remember aurally
8.	What is the duration of the training session that you can withstand without stress?	● 15–18 min ● 45 min ● 1.5 h ● I can withstand more than 2 h if I am allowed to move, rather than just sitting in one place ● Lying down, I can listen for as long as I like
9.	In the future, you expect to be employed:	● In the office among other employees ● Remotely as a freelancer ● Managing innovations ● I like to travel and do field research ● I do not want to work, I want to continue to study
10.	In the future, you expect to be employed (secondary):	● In the office among other employees ● Remotely as a freelancer ● Managing innovations ● I like to travel and do field research ● I do not want to work, I want to continue to study

## References

[1] Istomina N.L., Trubetskova E.G. From Haeckel to global ecology (through the view of two cultures). V Congress «Globalistics-2017». Moscow, Moscow State University. Russia, **2017**, 2. https://lomonosov-msu.ru/archive/Globalistics_2017/data/10131/uid160785_report.pdf.

[2] Montserrat D.-M., Parede M.R., Saren M. (2019). Improving Society by Improving Education through Service-Dominant Logic: Reframing the Role of Students in Higher Education. Sustainability.

[3] Loshkareva E., Luksha P., Ninenko I., Smagin I., Sudakov D. Skills of the Future: How to Thrive in the Complex New World. Global Education Futures. World Skills Russia. Report 2014–2017. **2017**, 93. https://worldskills.ru/assets/docs/media/WSdoklad_12_okt_eng.pdf.

[4] Loshchikhina A. (2015). High Reading Science.

[5] Bykova O., Martynova M., Siromaha V. (2018). The Problem of Continuity in the Development of Creative Critical Thinking in the System of Modern School and University Education in Russia. Sci. Res. Dev. Mod. Commun. Stud..

[6] Russia in Numbers 2018. Brief Statistical Bulletin. Moscow, Rosstat **2018**, 522. https://www.gks.ru/free_doc/doc_2018/year/year18.pdf.

[7] Universities in the Eurasian Educational Space/Editorial Team: Sadovnichiy, V. et al. Publishing house of the Moscow State University; Moscow, Russia: MAKS Press, **2017**, 392 (Series «Eurasian universities of the XXI century»). http://www.eau-msu.ru/files/eau_mono.pdf.

[8] Tong V.C.H. (2014). Geoscience Research and Education Innovations in Science Education and Technology.

[9] Nazareth A., Newcombe N.S., Shipley T.F., Velazquez M., Weisberg S.M. (2019). Beyond Small-Scale Spatial Skills: Navigation Skills and Geoscience Education. Cogn. Res. Princ. Implic..

[10] Dolphin G., Dutchak A., Karchewski B., Cooper J. (2019). Virtual Field Experiences in Introductory Geology: Addressing a Capacity Problem, but Finding a Pedagogical One. J. Geosci. Educ..

[11] Krylov S.A., Zagrebin G.I., Dvornikov A.V., Kotova O.I. (2019). Geoinformation technologies in the educational process of the department of cartography of MIIGAiK. Actual Quest. Educ..

[12] Site of the University MIIGAiK. http://www.miigaik.ru/eng/history.htm.

[13] Bykova O.P., Martynova M.A., Siromaha V.G. (2018). University Audience as A Glade of Meanings: To the Problem Statement. Dynamics Of Language And Cultural Processes In Modern Russia.

[14] Esteves H., Fernandes I., Vasconcelos C. (2015). A Field-Based Approach to Teach Geoscience: A Study with Secondary Students. Procedia Soc. Behav. Sci..

[15] Boyle A., Maguire S., Martin A., Milsom C., Nash R., Rawlinson S., Turner A., Wurthmann S., Conchie S. (2007). Fieldwork Is Good: The Student Perception and the Affective Domain. J. Geogr. High. Educ..

[16] Gilley B., Atchison C., Feig A., Stokes A. (2015). Impact of inclusive field trips. Nat. Geosci..

[17] Elkins J.T., Elkins N.M.L. (2007). Teaching Geology in the Field: Significant Geoscience Concept Gains in Entirely Field-Based Introductory Geology Courses. J. Geosci. Educ..

[18] Teasdale R., Ryker K., Bitting K. (2019). Training Graduate Teaching Assistants in the STEM education: Our Practices vs. Perceived Needs. J. Geosci. Educ..

[19] Gruzinov V.S. (2019). Educational field test sites: Problems and ways of development. Izvestia vuzov «Geodesy and Aerophotosurveying».

[20] Bykova O.P., Martynova M.A., Siromaha V.G. New educational paradigm—New requirements to the lecturer. Materials of the V All-Russian scientific-practical Conference with international participation *Humanitarian Technologies in the Modern World,* Ed. Goncharova, L.M.; Western branch of Russian Presidential Academy of National Economy and Public Administration Kaliningrad: Baltic Guard of the Ministry of Defense of Russia: Kaliningrad, Russia, **2017**, 99–103. http://www.zf.ranepa.ru/upload/documents/npk_mats/sbor_mat_conf_05_2017.pdf.

[21] Izhvanov Y.L. (2017). Scientific-educational computer networks. Past, present and development trends. Educ. Resour. Technol..

[22] Cliffe A.D. (2017). A Review of the Benefits and Drawbacks to Virtual Field Guides in Today’s Geoscience Higher Education Environment. Int. J. Educ. Technol. High. Educ..

[23] Kamińska D., Sapiński T., Wiak S., Tikk T., Haamer R., Avots E., Helmi A., Ozcinar C., Anbarjafari G. (2019). Virtual Reality and Its Applications in Education: Survey. Information.

[24] Stainfield J., Fisher P., Ford B., Solem M. (2000). International Virtual Field Trips: A New Direction?. J. Geogr. High. Educ..

[25] Litherland K., Stott T.A. (2012). Virtual Field Sites: Losses and Gains in Authenticity with Semantic Technologies. Technol. Pedagog. Educ..

[26] Mead C., Buxner S., Bruce G., Taylor W., Semken S., Anbar A.D. (2019). Immersive, Interactive Virtual Field Trips Promote Science Learning. J. Geosci. Educ..

[27] Jacobson A.R., Militello R., Baveye P.C. (2009). Development of Computer-Assisted Virtual Field Trips to Support Multidisciplinary Learning. Comput. Educ..

[28] Schiappa T.A., Smith L. (2019). Field Experiences in STEM education: A Case Study from a Multidisciplinary Geology and Geography Course. J. Geosci. Educ..

[29] Hesthammer J., Fossen H., Sautter M., Sæther B., Johansen S.E. (2002). The Use of Information Technology to Enhance Learning in Geological Field Trips. J. Geosci. Educ..

[30] Spencer E.W. (1990). Introductory Geology with a Field Emphasis. J. Geol. Educ..

[31] Johnson J.K., Reynolds S.J. (2005). Concept Sketches—Using Student- and Instructor-Generated, Annotated Sketches for Learning, Teaching, and Assessment in Geology Courses. J. Geosci. Educ..

[32] Hurst S.D. (1998). Use of ‘Virtual’ Field Trips in Teaching Introductory Geology. Comput. Stem Educ..

[33] Site of the Research Station RAS. http://www.gdirc.ru/en/.

[34] Bataleva E.A., Mukhamadeeva V.A. (2018). Complex electromagnetic monitoring of geodynamic processes in the Northern Tien Shan (Bishkek Geodynamic Test Area). Geodyn. Tectonophys..

[35] Bataleva E., Rybin A., Matiukov V. (2019). System for Collecting, Processing, Visualization, and Storage of the MT-Monitoring Data. Data.

[36] Site of the Chekhov Geopolygon MIIGAiK. http://www.miigaik.ru/sveden/objects/chekhovskiy/.

[37] Modin I.N., Jakovlev A.G., Bobachev A.A., Kulikov V.A., Khmelevskoj V.K. New place for MSU students field geophysical training—Alexandrovka. Proceedings of the 4th EEGS Meeting.

[38] Lygin I.V., Bulychev A.A., Gilod D.A., Sokolova T.B., Fadeev A.A. (2014). The results of gravity surveys at geophysical grounds in the Kaluga region. Moscow Univ. Geol. Bull..

[39] Site of the Company “Nord-West”. http://nw-geo.ru/alexandrovka/practices/.

[40] Siemens G., McGreal R., Kinuthia W., Marshall S. (2013). Massive open online courses: Innovation in education?. Open Educational Resources: Innovation, Research and Practice.

[41] Kuznetsov N.V. (2019). Online education: Key trends and barriers. E-Manage.

[42] Robinson A.C., Kerski J., Long E.C., Luo H., DiBiase D., Lee A. (2015). Maps and the geospatial revolution: Teaching a massive open online course (MOOC) in geography. J. Geogr. High. Educ..

[43] Mikeš D. (2015). Geoscience Education Is Outdated. S. Afr. J. Geol..

[44] Site of the Ministry of Science and Higher Education of Russian Federation. https://www.minobrnauki.gov.ru/ru/activity/digitalcouncil/digitalobr/grantsupport/.

[45] Site of the Modern Digital Educational Environment in Russia. http://neorusedu.ru/.

[46] National Online Educational Platform “Open Education”. https://openedu.ru/.

[47] The International Coursera Portal. https://www.coursera.org/.

[48] Beane R., McNeal K.S., Macdonald R.H. (2019). Probing the National Geoscience Faculty Survey for Reported Use of Practices That Support Inclusive Learning Environments in Undergraduate Courses. J. Geosci. Educ..

[49] Aptekman A., Kalabin V., Klintsov V., Kuznetsova E., Kulagin V., Yasenovets I. (2017). Digital Russia: A New Reality.

